# Reconfigurable Solid-state Dye-doped Polymer Ring Resonator Lasers

**DOI:** 10.1038/srep18310

**Published:** 2015-12-17

**Authors:** Hengky Chandrahalim, Xudong Fan

**Affiliations:** 1 Department of Biomedical Engineering, University of Michigan, 1101 Beal Ave., Ann Arbor, MI 48109, USA

## Abstract

This paper presents wavelength configurable on-chip solid-state ring lasers fabricated by a single-mask standard lithography. The single- and coupled-ring resonator hosts were fabricated on a fused-silica wafer and filled with 3,3′-Diethyloxacarbocyanine iodide (CY3), Rhodamine 6G (R6G), and 3,3′-Diethylthiadicarbocyanine iodide (CY5)-doped polymer as the reconfigurable gain media. The recorded lasing threshold was ~220 nJ/mm^2^ per pulse for the single-ring resonator laser with R6G, marking the lowest threshold shown by solid-state dye-doped polymer lasers fabricated with a standard lithography process on a chip. A single-mode lasing from a coupled-ring resonator system with the lasing threshold of ~360 nJ/mm^2^ per pulse was also demonstrated through the Vernier effect. The renewability of the dye-doped polymer was examined by removing and redepositing the dye-doped polymer on the same resonator hosts for multiple cycles. We recorded consistent emissions from the devices for all trials, suggesting the feasibility of employing this technology for numerous photonic and biochemical sensing applications that entail for sustainable, reconfigurable, and low lasing threshold coherent light sources on a chip.

Since the pioneering work back in the 1960s and 1970s^1^,^2^,^3^,^4^, solid-state dye-doped polymer lasers have found many applications in optical communication devices^5^, advanced nanoscale lasers^6^, biosensing^7^,^8^,^9^, and novel integrated photonic systems on-chip^10^. Recently, there has been a surge of research to explore innovative optical resonator designs as the feedback component for the lasers. Bragg gratings based devices such as distributed feedback (DFB) structures^11^,^12^,^13^,^14^,^15^,^16^,^17^,^18^,^19^,^20^ are the most commonly used cavity structures in solid-state dye lasers. These devices exhibit certain compelling characteristics, such as low pump thresholds, tunable wavelengths, and single-mode of operations. However, the manufacturing of these devices is not straightforward and rather expensive as electron-beam (e-beam) lithography to pattern the molds^13^,^15^,^17^ or holography lithographic technique^11^,^12^,^14^ is necessary. In addition, the laser emission is limited to one wavelength region determined by the emission band of the selected dye. Changing from one wavelength region (*i.e*., one dye) to another region (*i.e*., another dye) requires fabrication of new DFB structures. Photonic crystal based dye-doped polymer lasers have also been explored to exhibit single-mode emission^21^,^22^,^23^ and good repeatability^24^. Nevertheless, they share the same drawbacks as DFB lasers in terms of fabrication complexity, cost, and wavelength flexibility.

The micro-ring and micro-disk optical cavities that operate based on the whispering gallery modes (WGM) are recognized to have relatively high quality factors (*Q*-factors) even if they are manufactured on a chip^8^,^25^,^26^,^27^. They have a simple geometrical shape, small footprint, and ability to accommodate a broad range of lasing emission wavelengths. Solid-state micro-ring and micro-disk dye-doped polymer lasers that have compact sizes^28^,^29^ and single-mode of operations^30^,^31^ have been previously presented. Recently, dye-doped polymer lasers based on WGM microgoblets for sensing applications were demonstrated^7^,^8^,^9^. A large array of microgoblet lasers can be fabricated in parallel by a single step of Deep-UV (DUV) lithography, followed by thermal reflow to form the goblets. Impressively, Bog *et al.* demonstrated the multiplexed deposition of different functionalization coatings on neighboring goblet structures^7^,^9^. While this method shows the great potential to manufacture solid-state lasers economically^8^, it lacks the accuracy to define the final shape and size of the ring resonators after the reflowing process.

In general, the polymer host materials of the solid-state dye lasers built to date naturally have a modest mechanical robustness, which is vulnerable to mechanical deformations, temperature fluctuations, and chemical reactions. Therefore, operating these polymer–based lasers under certain environmental conditions is challenging. More importantly, a full fabrication process of new devices is required once the device lifetime ends or radiations of different wavelengths are desired. A successful launch of solid-state dye lasers as practical submodules in photonic networks requires a simple, rapid, and economical fabrication process that can reliably mass-produce the lasers with flexibility in lasing wavelength, which can be configured virtually at any time. Ideally the solid-state dye lasers should be refillable systems that do not frequently require replacements from the newly fabricated devices. From the device application perspective, a laser host that has a high mechanical strength, broad chemical compatibility, and good thermal stability is greatly favorable. Several attempts have been reported to coat or dope the gain medium on the outer surface of a solid ring resonators on-chip^32^,^33^,^34^. However, such methods pose a limitation to precisely define the final dimensions of the resonators and/or have difficulty in controlling the gap between the two rings in a coupled ring resonator system.

Here, we report on the design, fabrication, and demonstration of solid-state ring lasers made of fused-silica and refillable by the reconfigurable dye-doped polymer that meets the aforementioned requirements (see Fig. 1). We built the devices with a simple single-mask standard lithography, followed by a dry reactive ion etching, dicing, and deposition of dye-doped polymer. We employed 3,3′-Diethyloxacarbocyanine iodide (CY3), Rhodamine 6G (R6G), and 3,3′-Diethylthiadicarbocyanine iodide (CY5) dyes as the gain media for our solid-state lasers. First, several dye-doped polymer single-ring resonators on the same chip were excited to demonstrate multiple lasers on a single chip. Next, the single optical ring resonators with CY3, R6G, and CY5-doped SU-8 were at 480, 530, and 570 nm, respectively, and achieved multimode lasing centered around 540, 590, and 770 nm. The lowest recorded lasing threshold is ~220 nJ/mm^2^ per pulse for the multimode single-ring cavity with R6G-doped SU-8. A single-mode lasing from a coupled-ring resonator system with the lasing threshold of ~360 nJ/mm^2^ was demonstrated through the Vernier effect. Finally, the renewability of the dye-doped polymer was tested by removing and re-depositing the dye-doped polymer on the same resonator hosts for multiple cycles. We recorded consistent laser emissions from the tested devices for all trials. Our fabrication process enables a durable solid-state laser platform that can be produced rapidly, reliably, and economically at the wafer level with a standard photolithography. The gain media in our process are deposited on mechanically, thermally, and chemically inert fused-silica host resonators, thereby providing excellent protective framework to the emitting polymer cores.

## Methods

**Figure 1:**
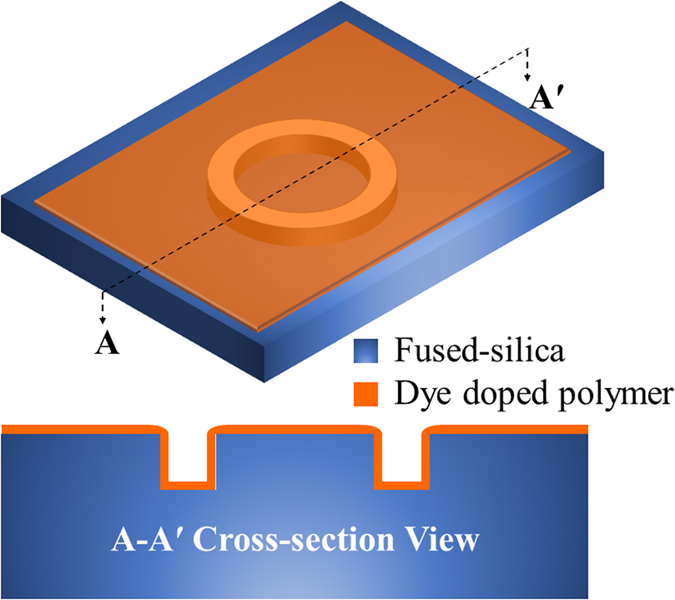
3-D and cross-sectional schematic of the fused-silica ring resonator coated with a gain medium doped high refractive index polymer.

The solid-state ring resonator lasers in this work comprised primarily of the fused-silica ring resonator hosts and the dye-doped high refractive index (>1.46) polymer deposited on it. The top and cross-sectional views of the device are illustrated in Fig. 1. The single-ring resonator was designed to have inner and outer radii and depth of 110, 150, and 12 μm, respectively. In order to achieve single-mode laser operation, we utilized the Vernier effect, in which two ring resonators of slightly dissimilar sizes hence the free spectral ranges (FSRs) are coupled to each other to suppress the side modes^31^,^35^,^36^,^37^. In our current coupled-ring system, each ring had an outer radius of 150 and 145 μm, respectively. The coupling distance between the two rings was designed to be 1 μm.

**Figure 2:**
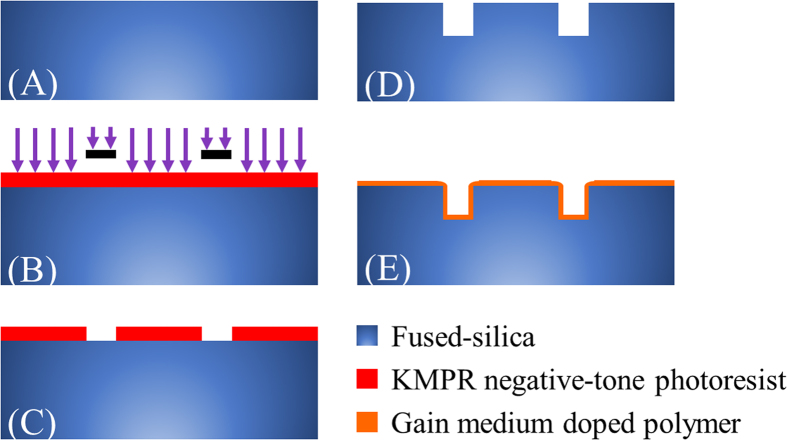
The fabrication process flow of the ring resonators: (**A**) 500 μm thick fused-silica wafer. (**B**) 14 μm thick KMPR negative-tone photoresist was spin-coated on the top of the fused-silica wafer and exposed to the UV light. (**C**) The developed photoresist on the fused-silica wafer. (**D**) The fused-silica wafer after a dry-etching process by using a reactive-ion etcher. (**E**) 5 μm thick layer of gain medium doped polymer was deposited and cured on the surface of the cavity.

**Figure 3:**
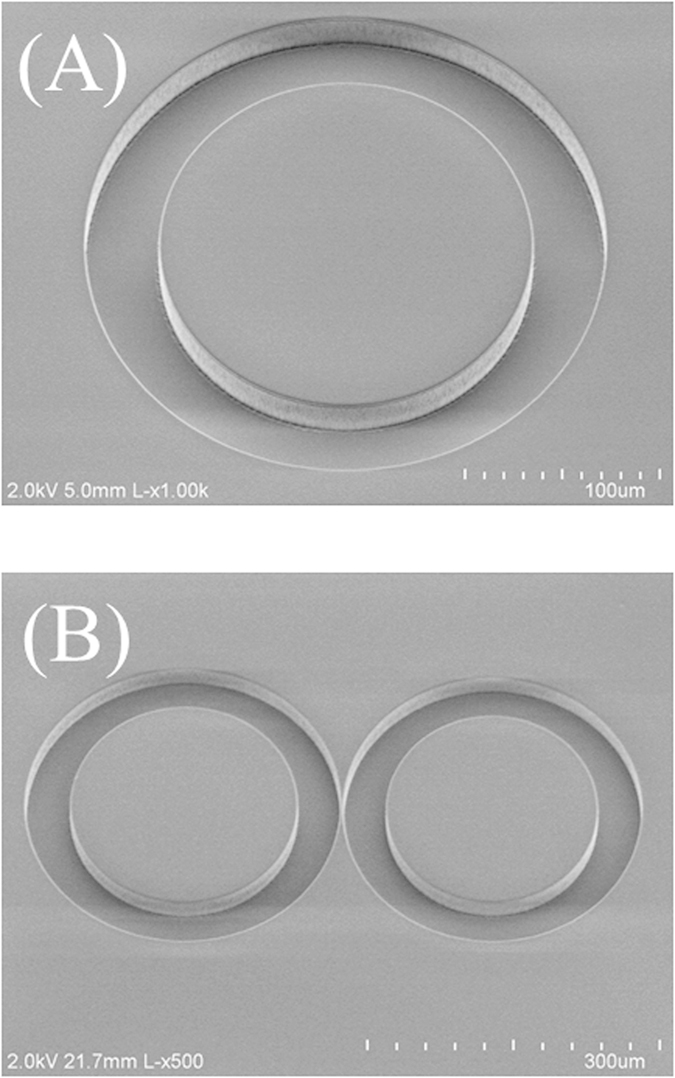
Scanning microscope images of the fabricated ring resonators: (**A**) Single ring resonator with inner and outer radii and depth of 110, 150, and 12 μm respectively. (**B**) Coupled ring resonators with an outer radius of 150 μm and 145 μm, respectively. Both ring resonators are 40 μm wide and 12 μm deep. The coupling distance between the two rings is approximately 1 μm.

The solid-state ring cavity structures were fabricated with a wafer level single-mask bulk micromachining process as outlined in Fig. 2. A 100 mm diameter fused-silica wafer was first cleaned in a Piranha solution (H_2_SO_4_ + H_2_O_2_) at 60 °C for 10 minutes. Next, the wafer was spin-coated with a 14 μm thick negative-tone photoresist (KMPR) and soft-baked at 100 °C for 6 minutes. A Suss MicroTec MA/BA-6 contact aligner was used to expose the wafer with the UV light. The wafer was then baked at 100 °C for 3 minutes and developed with a TMAH (Tetramethylammonium hydroxide) based photoresist developer. A reactive ion glass etcher was used to etch the fused-silica wafer. The chamber pressure, coil power, and platen power were set at 4 mTorr, 1400 W, and 300 W, respectively. The gasses used in this etching process were C_4_F_8_ and He with flow rates of 10 and 174 sccm, respectively. After the etching process, the wafer was diced into 1 cm×1 cm chips by using ADT 7100 dicing saw. The scanning electron (SEM) images of the fabricated single and coupled-ring resonators prior to polymer deposition are shown in Fig. 3. Finally, a 5 μm thick layer of dye-doped polymer was spin-coated and cured on the diced fused-silica chips.

**Figure 4:**
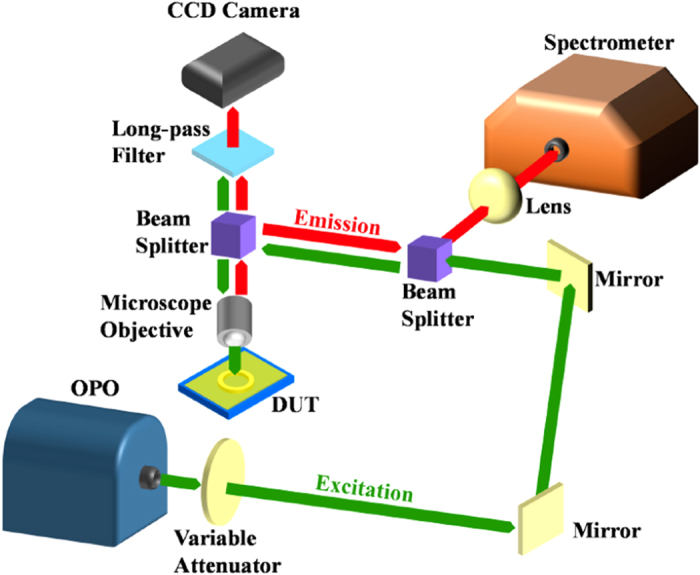
Schematic of the measurement setup.

Three types of dyes (CY3, R6G, and CY5) were first dissolved in ethanol (10 mM dye concentration for each solution) and subsequently mixed with SU-8 at a ratio of 10% (ethanol + dye) to 90% (SU-8) in the ultrasonic bath for 60 minutes. Each solution was then spin-coated onto the fused-silica chips that already have the ring cavity hosts patterned on them and baked for 30 minutes at 95 °C to evaporate the solvent and cure the polymer. The solid-state ring resonators that support the WGM were formed by the dye-doped polymer with a higher refractive index (1.6) than that of the surrounding fused-silica (1.46). Since the cured polymer is enclosed by a mechanically robust circumambient fused-silica, thus the conventional process of cross-linking the polymer with the UV light followed by post-baking is unnecessary. The absence of the polymer cross-linking process allows for easy removal of the polymeric gain media from the ring channels and re-deposition of alternative dye-doped polymers with different emission wavelengths as desired. The solid-state dye lasers were tested and characterized according to the measurement setup outlined in Fig. 4. Nanosecond pulses from an optical parametric oscillator (OPO, repetition rate = 20 Hz, pulse width = 5 ns) were transmitted through the variable attenuator and steered towards the beam splitter by two mirrors. The transmitted light went to another beam splitter and was focused to a spot area of 0.03 mm^2^ on the sample by using a microscope objective. The emitted laser beam from the cavity was divided by the beam splitter and directed towards the CCD camera and the spectrometer (Horiba iHR550).

## Results

**Figure 5:**
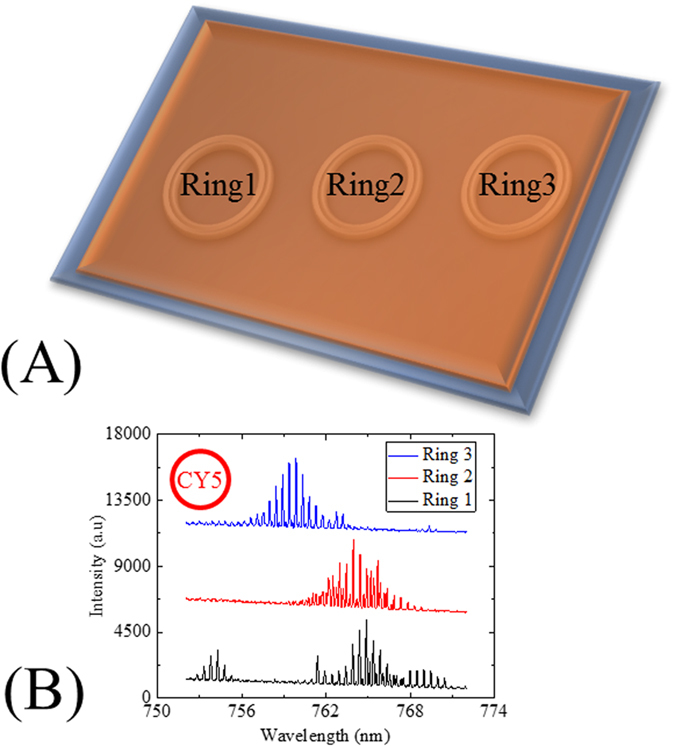
(**A**) A schematic of multiple dye-doped polymer single-ring resonators on a chip. (**B**) Lasing spectra of three different CY5-doped SU-8 single-ring resonator lasers on the same chip. The spot size of the OPO pumping laser beam was approximately 0.03 mm^2^. A 1200 g/mm grating was used to record the entire discernable multimode laser emissions. The three single-ring cavity lasers were excited with 10 μJ/mm^2^ at 570 nm. All spectra are vertically shifted for clarity.

First, we realized multiple lasers on the same chip with a single-mask one parallel lithography step. Three CY5-doped SU-8 single-ring lasers on the same chip as illustrated in Fig. 5(A) were excited independently by the OPO at 570 nm wavelength. Figure 5(B) plots the multimode laser emission spectra of three different CY5-doped SU-8 single-ring lasers on the same chip with 10 μJ/mm^2^ pump intensity. It should be emphasized that several fabrication methods, especially those applied to realize Bragg gratings and photonic crystal based cavities, require serial writings by e-beam lithography, which can be very time consuming and lead into astronomical fabrication costs when large arrays of devices on the substrate are needed. In contrast, the fabrication method that we developed here enables the realization of a large number of devices on a chip with reliable performance at low-cost in a timely manner.

**Figure 6:**
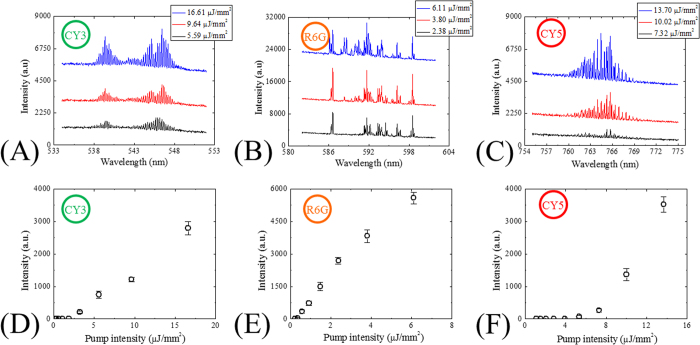
Lasing spectra of (**A**) CY3-doped SU-8, (**B**) R6G-doped SU-8, and (**C**) CY5-doped SU-8 single ring resonator lasers. All spectra are vertically shifted for clarity. (**D**–**F**) Spectrally integrated laser outputs (542–548 nm for CY3-doped SU-8, 586–601 nm for R6G-doped SU-8, 760–770 nm for CY5-doped SU-8) after fluorescence background removal as a function of the pump intensity extracted from (**A**–**C**). The lasing thresholds for CY3, R6G, and CY5-doped SU-8 are approximately 1.9, 0.2, and 3.9 μJ/mm^2^, respectively. Error bars are obtained with 3 measurements.

Additionally, our solid-state dye lasers offer a wide range of wavelength selections across the wafer due to the spectral availability of the WGMs. Here we used CY3, R6G, and CY5-doped SU-8 single-ring lasers as model systems, which were excited by the OPO at 480, 530, and 570 nm, respectively. The multimode lasing outputs centered around 540, 590, and 770 nm were observed from those lasers, as shown in Fig. 6 (A–C) with different the pump intensities. Figure 6(D–F) display the spectrally integrated laser emissions after fluorescence background removal as a function of the pump intensity. The lowest recorded lasing threshold is ~220 nJ/mm^2^ per pulse for the R6G-doped SU-8 single-ring cavity laser, marking the lowest threshold shown by solid-state dye-doped polymer lasers fabricated with a standard lithography process on a chip.

The commonly known manufacturing processes of polymer lasers, regardless the types of cavities, usually permit only one homogeneous deposition of organic gain media across the same substrate for each deposition step. This imposes a limitation on the choices of the center wavelengths at which the lasers operate. The gain media in our process are deposited on fused-silica chips after the wafer dicing process. Consequently, a large variety of gain media with different emission ranges can be deposited independently on these chips.

**Figure 7:**
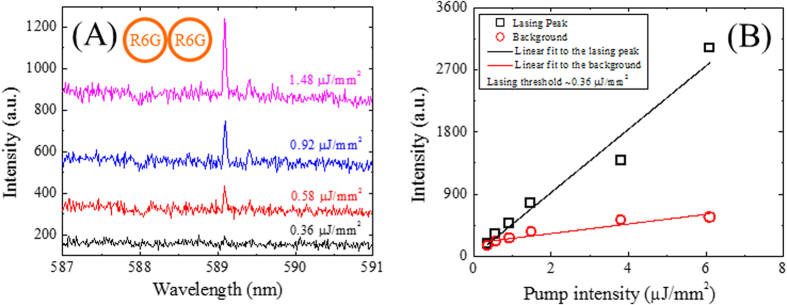
(**A**) Single-mode emissions of a R6G-doped SU-8 coupled-ring resonator laser. A 2400 g/mm grating was used to achieve high spectral resolution. The full-width-at-half-maximum is approximately 0.03 nm, limited by the instrument’s spectral resolution. Spectra are vertically shifted for clarity. (**B**) Lasing peaks as a function of the pump intensity extracted from (**A**). The lasing threshold is approximately 0.36 μJ/mm^2^.

The single-mode emission feature is essential in many applications, such as laser spectroscopy, laser metrology, and biomolecular sensing. The WGM single-ring resonator lasers intrinsically have multimode lasing emissions due to their narrow FSRs, which is a major drawback as compared to the DFB based lasers that normally have a single laser mode. Here we designed and fabricated coupled-ring optical cavities on the same chip and used the Vernier effect to produce single-mode laser emissions. The lasing spectra of the coupled-ring cavity filled with R6G-doped SU-8 at various pump intensities are plotted in Fig. 7(A). The single-mode emission at 589 nm started to emerge with the resolution limited linewidth of 0.03 nm. The corresponding lasing threshold curve is plotted in Fig. 7(B), showing a lasing threshold of 360 nJ/mm^2^. It is noted that the lasing threshold of the coupled-ring cavity is slightly higher than the single-ring cavity. This might be due to the fact that the coupled-ring cavity experienced more light scatterings from the two ring constituents.

**Figure 8:**
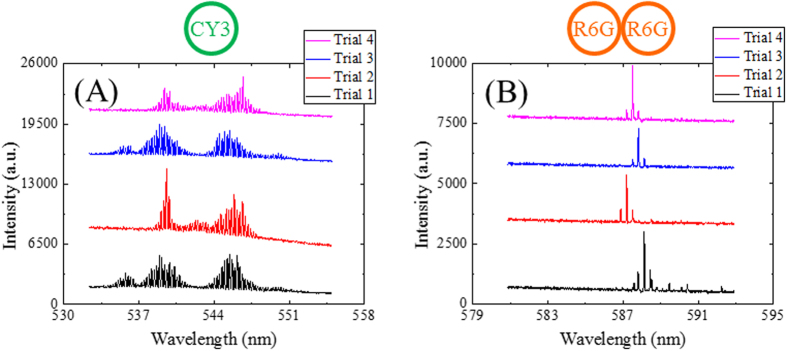
Consistent laser emissions are recorded from the same ring resonator hosts after four cycles of removals and re-depositions of dye-doped polymer. (**A**) Emission spectra of a CY3-doped SU-8 single ring resonator laser with a 16 μJ/mm^2^ pump intensity. A 1200 g/mm grating was used to capture the entire detectable laser emissions. (**B**) Emission spectra of an R6G-doped SU-8 coupled-ring resonator laser with a 6 μJ/mm^2^ pump intensity. A 2400 g/mm grating was used to achieve high spectral resolution. All spectra are vertically shifted for clarity.

One of the major advantages of our dye-doped polymer laser over the existing polymer lasers is its simple regeneration capability, which can easily be achieved by removing and re-depositing identical dye-doped polymer on the same resonator hosts for multiple cycles. We first performed the dye laser renewability experiments on the single-ring cavity laser. The CY3-doped SU-8 on the chip was removed by Remover PG solution (MicroChem) and re-deposited for multiple cycles. The pump intensity of 16 μJ/mm^2^ at 480 nm was used to excite the gain medium after active polymer re-deposition on the resonator in all experiments. Consistent multimode emission spectra were recorded at around 540 nm center wavelength as shown in Fig. 8(A).

Next, we performed similar experiments to justify the renewability of dye laser in the coupled-ring laser. We performed multiple cycles of removal and deposition of R6G-doped SU8 on the identical coupled-ring cavity. Given the rich WGM spectrum for the cavity dimensions that we have (FSR ~0.23 nm), the Vernier becomes less effective in suppressing the side modes at high pump intensities, leading to the emergence of the neighboring side modes. However, the pump intensity at which the side modes starts to appear can be used as an additional parameter to measure consistency of our ring resonator lasers. In the experiment, we increased the pump intensity (at 530 nm) until the side modes (1 FSR away from the central laser line) started to emerge at 6 μJ/mm^2^ (~17× the lasing threshold) and used the same pump intensity to excite the cavity under test. We recorded consistent laser emissions between 587 to 588 nm from the same coupled-ring cavity laser after active polymer re-deposition cycles as shown in Fig. 8(B). In addition, the neighboring modes started to appear in the spectra at 6 μJ/mm^2^ pump intensity for all trials, confirming the repeatability of the laser performance.

## Discussion

We have successfully designed, fabricated, and achieved multimode and single-mode wavelength configurable on-chip solid-state dye-doped polymer ring resonator lasers. Such lasers offer advantageous features compared to the existing solid-state dye lasers, such as a large array of excitable emissions from a single chip, low lasing thresholds, inherent mechanical robustness, flexible wavelength selection across the wafer, and the regeneration of the emitting polymer cores. Particularly, in comparison with the popular DFB based polymer lasers, which require expensive fabrication techniques and are less flexible in accommodating dyes of various emission wavelengths, the current devices can be mass-produced rapidly, reliably, and economically at the wafer level. In comparison with the design in which the gain medium is placed on the outer surface of a ring resonator, the present method (coating on the inner surface) allows us to precisely control the final ring resonator dimensions and the gap between the two coupled ring resonators. Therefore, the solid-state ring resonator lasers studied in this work can be an attractive candidate for various photonics applications that require sustainable, reconfigurable, dependable, and low-cost coherent light sources on a chip. In addition to dyes, fluorescent conjugate polymers^38^, fluorescent proteins^39^, quantum dots^32^,^40^,^41^, rare-earth ions^34^ can be explored as gain media in the future. Finally, the same structures, in particular, the coupled ring resonator can be adapted to accommodate the liquid gain medium using 3-D microfluidics as shown in our recent work^42^.

## Additional Information

**How to cite this article**: Chandrahalim, H. and Fan, X. Reconfigurable Solid-state Dye-doped Polymer Ring Resonator Lasers. *Sci. Rep.* **5**, 18310; doi: 10.1038/srep18310 (2015).
